# Super-refractory Status Epilepticus and Cerebellar Involvement Following High-Voltage Electrical Injury

**DOI:** 10.7759/cureus.77472

**Published:** 2025-01-15

**Authors:** Ragasivamalini B, V Balambighai, Stephen A Sureshkumar

**Affiliations:** 1 Neurology, Sree Balaji Medical College and Hospital, Chennai, IND

**Keywords:** cerebellar involvement, electrical injury, myoclonic jerks, super-refractory status epilepticus, young female

## Abstract

Electrocution injuries can impact any organ or system in the body. The symptoms vary widely, from immediate cardiac arrest due to high-voltage current to mild discomfort caused by low-voltage current. There are four main types of electric injuries: flash, flame, lightning, and true direct injuries. Among these true and flame electric injuries are those that involve the body and have distinct entrance and exit sites.

Immediate effects on the central nervous system following a high-voltage injury may include loss of consciousness, confusion, seizures, and poor recall. However, seizures as a complication of electric brain injury are rarely reported. Central syndromes, including cerebellar syndromes, can be a result of both traumatic events and the mechanical effects of current flow, which may lead to an increased risk of morbidity.

Complications related to the peripheral nervous system, cognitive impairment, and psychological effects are less commonly reported after electric injuries. In this context, we describe a rare case of electrical injury accompanied by unusual neurological complications.

## Introduction

Electrical injuries pose a substantial global health threat, with significant morbidity and mortality. Neurological complications of electrical injuries often present the most severe and enduring consequences. The passage of electrical current through the body can inflict immediate and long-lasting damage on the nervous system, resulting in a spectrum of neurological manifestations. These complications can vary widely in severity, ranging from subtle sensory disturbances to devastating and irreversible disabilities such as paralysis, cognitive impairment, and seizures.

Electrical injuries are categorized as low-voltage or high-voltage, with the type of current (alternating or direct) significantly influencing the severity of neurological damage. The pathways traversed by the electrical current within the body also play a crucial role in determining the specific neurological complications. Fundamentally, the neurological effects of electrical injury stem from the passage of electrical current through the body. As the current travels through tissues, it generates heat, exerts mechanical forces, and induces electromagnetic effects. These factors can lead to cellular injury, particularly within the nervous system. The extent of damage is influenced by several factors, including voltage, current intensity, duration of contact, and the specific pathway traversed by the electrical current.

The effects of electrical injury are often categorized as either direct or indirect. The current itself causes nerve cell depolarization, leading to immediate dysfunction. This can affect both the central nervous system (CNS) and the peripheral nervous system (PNS). In high-voltage injuries, the direct effects are more pronounced due to the greater energy passing through the body.

Indirect effects of electrical injury result from the body's response to it, such as hypoxia, ischemia, or inflammation, which can indirectly affect nerve function. The damage to blood vessels, for example, may lead to ischemia in nerve tissues, exacerbating neurological injury.

In this article, we describe a rare case of electrical injury in a young female with uncommon neurological complications that were identified and managed appropriately, resulting in a good neurological recovery.

## Case presentation

**Figure 1:**
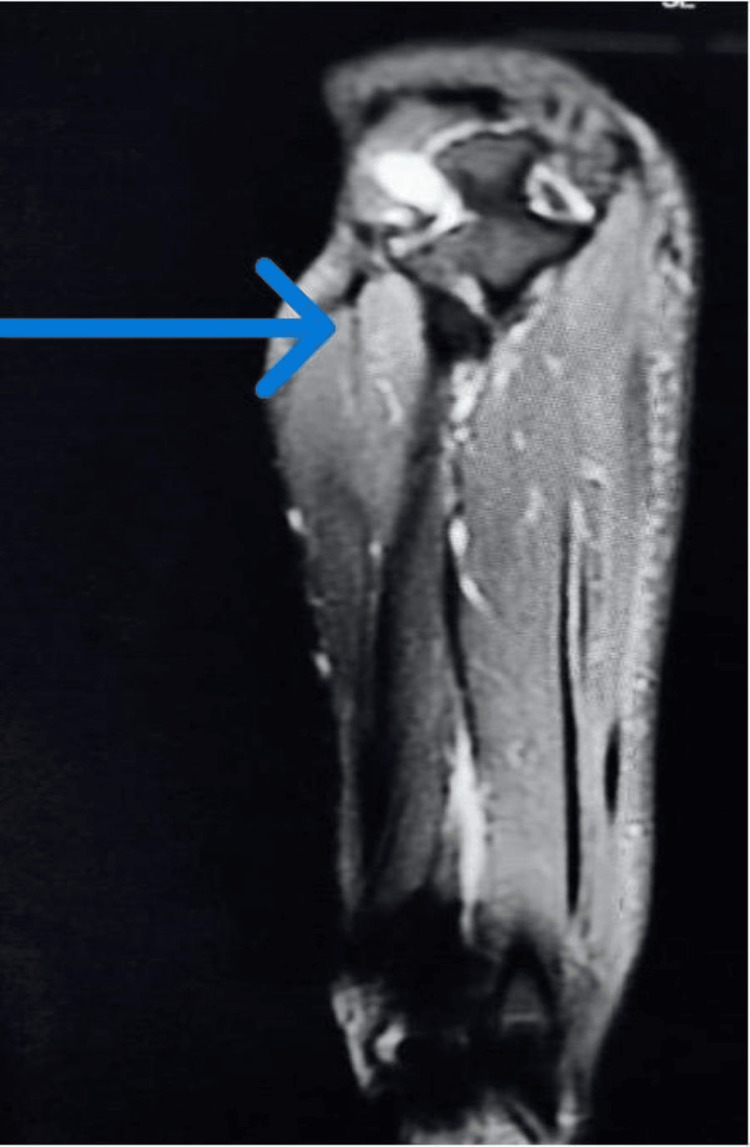
MRI right forearm Hyperintense edematous signal in subcutaneous plane, cubital fossa region surrounding underlying vessels, and mild joint effusion in elbow.

A young female, in her early twenties with no comorbidities, presented to the Neurology Outpatient Department (OPD) with acute onset of giddiness, chest discomfort, pain, and swelling of her right upper limb following high-voltage electric shock injury from a common water dispenser in her right hand. She was found unconscious and regained consciousness after 10 minutes. On examination, the patient was conscious and oriented and had excruciating pain and swelling of the right upper limb and left-hand fingers, and both upper limb movements were restricted (right > left). Her serum total creatine phosphokinase levels were normal. MRI of the right forearm showed a hyperintense edematous signal in the subcutaneous plane of the cubital fossa region surrounding underlying vessels and mild joint effusion at the right elbow (Figure 1). The MRI of the brachial plexus was normal. After controlling the pain for three days, a nerve conduction study of both upper limbs was done, which showed severe motor (axonal) neuropathy in the right median nerve and both ulnar nerves. Complete CNS evaluation, including electroencephalogram (EEG) and MRI brain, was normal. She was managed by a multidisciplinary team consisting of neurologists, intensivists, psychiatrists, and physiotherapists.

**Figure 2:**
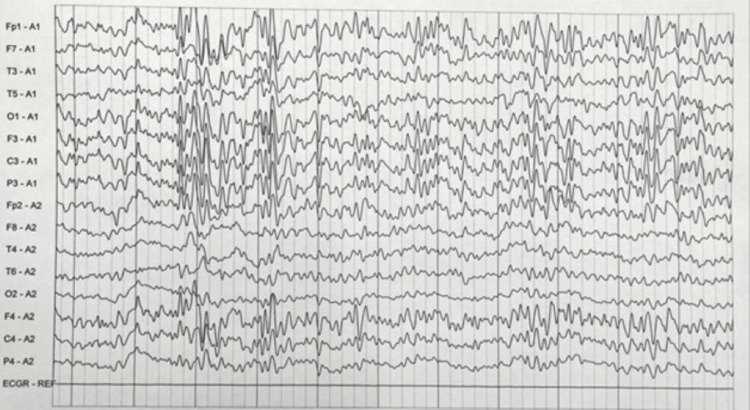
Electroencephalogram Findings showing abnormal bilateral epileptiform discharges.

On the 27th day of admission, she developed right focal seizures followed by generalized tonic-clonic seizures (GTCS), which progressed to super-refractory status epilepticus on day 28, necessitating endotracheal intubation and mechanical ventilation. She was on treatment with intravenous (IV) sodium valproate 1.5 g/day, intravenous levetiracetam 2 g/day, intravenous fosphenytoin 450 mg/day, intravenous lacosamide 200 mg/day, and oral perampanel 8 mg/day. She was on ventilatory support with five antiepileptic drugs along with midazolam infusion at 2 mg/hr, which was escalated to 4 mg/hr. Since seizures persisted, midazolam infusion was stopped, and propofol infusion was started, followed by sodium thiopentone infusion. Continuous EEG recordings showed generalized slow waves suggestive of global cerebral dysfunction (Figure 2). Repeat MRI brain with contrast study was normal.

On the 30th day of admission, CSF analysis was done, which showed clear liquid with no cells, protein of 42 mg/dl, glucose of 68 mg/dl (simultaneous blood glucose level 124 mg/dl), and CSF culture was sterile. The CSF's IgG index was recorded at -0.2. CSF meningoencephalitis and autoimmune panels were negative. Considering the physiological alterations caused by the passage of electric current altering the brain electrophysiology, and to prevent irreversible neurological damage, a high dose of intravenous methylprednisolone, 1 g/day, was given for a period of five days. Following this treatment, seizures reduced in frequency, and overall clinical condition began to improve. Oral prednisolone 1 mg/kg/day was started and tapered over a period of four weeks and stopped.

On the 31st day of admission, the patient developed myoclonic jerks, which led to the discontinuation of IV fosphenytoin while other antiepileptic drugs were continued. She was extubated after a seizure-free period of 24 hours clinically.

By the 36th day of admission, she developed cerebellar signs, including dysarthria, titubation, truncal ataxia, and a positive Romberg's sign. A repeat MRI of the brain and a whole spine screening showed normal results, and nerve conduction studies (NCS) indicated significant improvement in the motor action potential (MAP), compound muscle action potential (CMAP), and sensory nerve action potential (SNAP) across all four limbs.

During her hospitalization, the patient also experienced pseudo-seizures, which were effectively managed. She remained seizure-free for one week, showed improved muscular strength, and her cerebellar signs improved. Physiotherapy continued, and she was eventually discharged.

**Figure 3:**
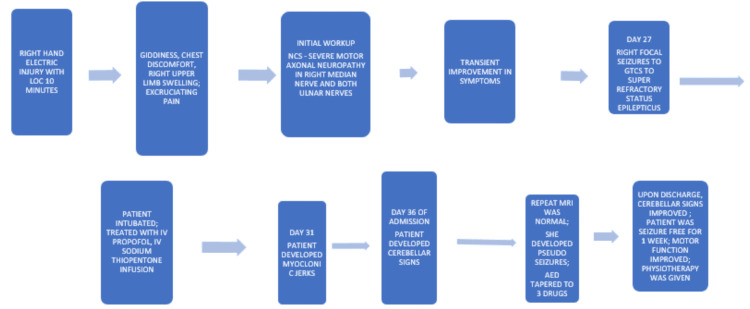
Sequence of clinical events after the electrical injury in our patient LOC: level of consciousness; NCS: nerve conduction studies; GTCS: generalized tonic-clonic seizures Image is created by the author.

During follow-up over the past year, her antiepileptic drugs (AEDs) regimen was titrated to three medications, and she has remained seizure-free for the entire year without any new neurological symptoms. She has returned to work and is under close follow-up. This case highlights the importance of multidisciplinary care and close follow-up in managing complex neurological complications following severe electrical injuries (Figure 3).

## Discussion

Electric injuries can impact any organ or system in the body. The symptoms vary widely, from immediate cardiac arrest due to high-voltage current to mild discomfort caused by low-voltage current. There are four main types of electric injuries: flash, flame, lightning, and true direct injuries. True and flame electric injuries involve the body and have distinct entrance and exit sites. [1] 

Immediate effects on the central nervous system following a high-voltage injury can include loss of consciousness, confusion, seizures, and impaired memory. Seizures are seen immediately in some patients with electric injury. However, the development of super-refractory status epilepticus and cerebellar involvement, which are seen in our patient as an immediate complication, is quite rare and scarcely reported. Central nervous system syndromes, including cerebellar syndromes, can result from both traumatic events and the mechanical effects of electrical current flow, which may lead to an increased risk of morbidity. Complications related to the peripheral nervous system, cognitive impairment, and psychological effects are less commonly reported after electric injuries [2].

Electrical injuries pose unique challenges due to the multifactorial nature of the damage they cause. Electric injuries can lead to a range of neurological complications that occur both immediately and later on. This case shows the complexity and severity of neurological complications due to high-voltage electrical injuries. Understanding these complications is crucial for providing effective treatment and ensuring optimal recovery.

Immediate neurological complications 

Loss of sensation, motor weakness, or paralysis in the affected limb can occur due to the direct impact of electric current on peripheral nerves and muscles [3]. Patients may experience intense pain and sensory symptoms such as tingling, numbness, and burning sensations following an electric injury, which may indicate underlying nerve damage [4]. Additionally, seizures may occur immediately after an electric shock, resulting from cortical irritation or direct brain injury. This recognized immediate complication is often attributed to the electrical disruption of neuronal activity.

High-voltage electrical injuries can also induce cardiac arrhythmias, including ventricular fibrillation, which can be life-threatening and require immediate resuscitation and monitoring. Furthermore, muscle damage resulting from these injuries can lead to rhabdomyolysis, which releases myoglobin into the bloodstream, causing myoglobinuria and potentially resulting in acute kidney injury [5].

Late neurological complications

Persistent neuropathic pain and chronic sensory disturbances, such as numbness and paresthesia, can develop in the affected limb long after an initial injury. These symptoms result from incomplete nerve regeneration or permanent nerve damage, which necessitates long-term pain management [6]. Additionally, muscle weakness and atrophy may persist due to irreversible damage to the peripheral nerves or muscle tissues. Rehabilitation and physical therapy are crucial for managing these complications, although some patients may never fully regain their function. Electrical injuries can lead to prolonged motor deficits and muscle weakness, requiring extensive rehabilitation efforts [7].

Patients who experience immediate seizures due to electrical injuries are at risk of developing post-traumatic epilepsy, which requires ongoing antiepileptic medication and close monitoring. Post-traumatic epilepsy is a recognized late complication in patients with electrical injuries and is often associated with significant long-term morbidity [8]. The mechanisms are unknown, and the hypothesis of direct damage or delayed indirect effect on the nervous system causing neuronal ischemia, chronic inflammation inducing electrostatic separation of tissues and electroporation of cells, cellular loss, and functional deterioration has been postulated [9]. 

Late neurological complications may include cognitive and psychological effects, such as memory impairment, anxiety, depression, and post-traumatic stress disorder (PTSD). The psychological and cognitive sequelae of electrical injuries can be profound, leading to long-lasting effects on the patient’s mental health and daily functioning. Additionally, delayed neurological syndromes, such as cerebellar ataxia or autonomic dysfunction, may occur weeks or months after the initial injury. These delayed neurological syndromes can complicate the recovery process and pose additional management challenges [10].

Scarring from burns and surgical interventions can result in contractures, which restrict the range of motion and function of the affected limb, necessitating physical therapy or surgical correction [11]. Furthermore, the involvement of anterior horn cells (AHCs) has been noted as a long-term complication of electrical injury.

## Conclusions

Electrical injuries can result in various neurological complications that impact both the central and peripheral nervous systems. Our patient exhibits rare and potentially life-threatening complications, including seizures, super-refractory status epilepticus, and cerebellar involvement. These complications are seldom reported in cases of electrical injuries. 

The multidisciplinary approach to the patient's care, which included neurologists, intensivists, psychiatrists, and physical therapists, was crucial in addressing the various complications she faced. The patient's recovery, marked by being seizure-free for one year and successfully returning to work, highlights the importance of long-term follow-up and careful management with antiepileptic drugs. Regular monitoring and appropriate adjustments to medication are essential for maintaining seizure control and enhancing the quality of life for patients recovering from electrical injuries.
